# Supplementary material for: Skipping Breakfast for 6 Days Delayed the Circadian Rhythm of the Body Temperature without Altering Clock Gene Expression in Human Leukocytes
Source: Nutrients. 2020 Sep 12;12(9):2797. doi: 10.3390/nu12092797 (PMC7551061; doi:10.3390/nu12092797)
Supplement: Supplementary file 1 [file nutrients-12-02797-s001.pdf]

nutrients MDPI

Supplementary Table 1. Primer sequences for quantitative real-time PCR.

Gene	Accession No.	Forward primer (5′ -->3′)	Reverse Primer (5′ -->3′)
CLOCK	NM_004898.3	ACTCCACCCAGGCAGCATTTA	TGGGACATGCCTTGTGGAA
BMAL1	NM_001030272.1	CGGAGTCGATGGTTCAGTTTCA	AGCATGCTGTCCATGCTGTG
PER1	NM_002616.3	ACAAGCAAATACTTTGGCAGCATC	CCTGCTTCAGCACAGAGGTCA
PER2	NM_022817.3	GTGCCAAGCAGTTGACTTAATAGGA	CCTGTGTAAGCACACACACTAAAGA
PER3	NM_016831.2	GCGCACTCATATATGCTAAGCCTTC	CTGCGACAAGCACCAGTTTCA
CRY1	NM_004075.4	CCTCCAATGTGGGCATCA	GAATCACAAACAGACGGGAGTTTA
CRY2	NM_021117.3	CATCATGAAGATGGCCAAGGA	GGCCTGAAAGCGCTTGTATGTAA
NR1D1	NM_021724.5	GTGAAGACATGACGACCCTGGA	GGAGCCACTGGAGCCAATGTA
NR1D2	NM_005126.4	AGATGTCAGCAATGTCGCTTCA	GCTTAGGAATACGACCAAACCGAAC
DBP	NM_001352.4	GCACACCTCTCGGTGCAGAT	GATCAAGCGGTCGGCTCTTT
TBP	NM_003194.5	TGCTGCGGTAATCATGAGGATA	TGAAGTCCAAGAACTTAGCTGGAA

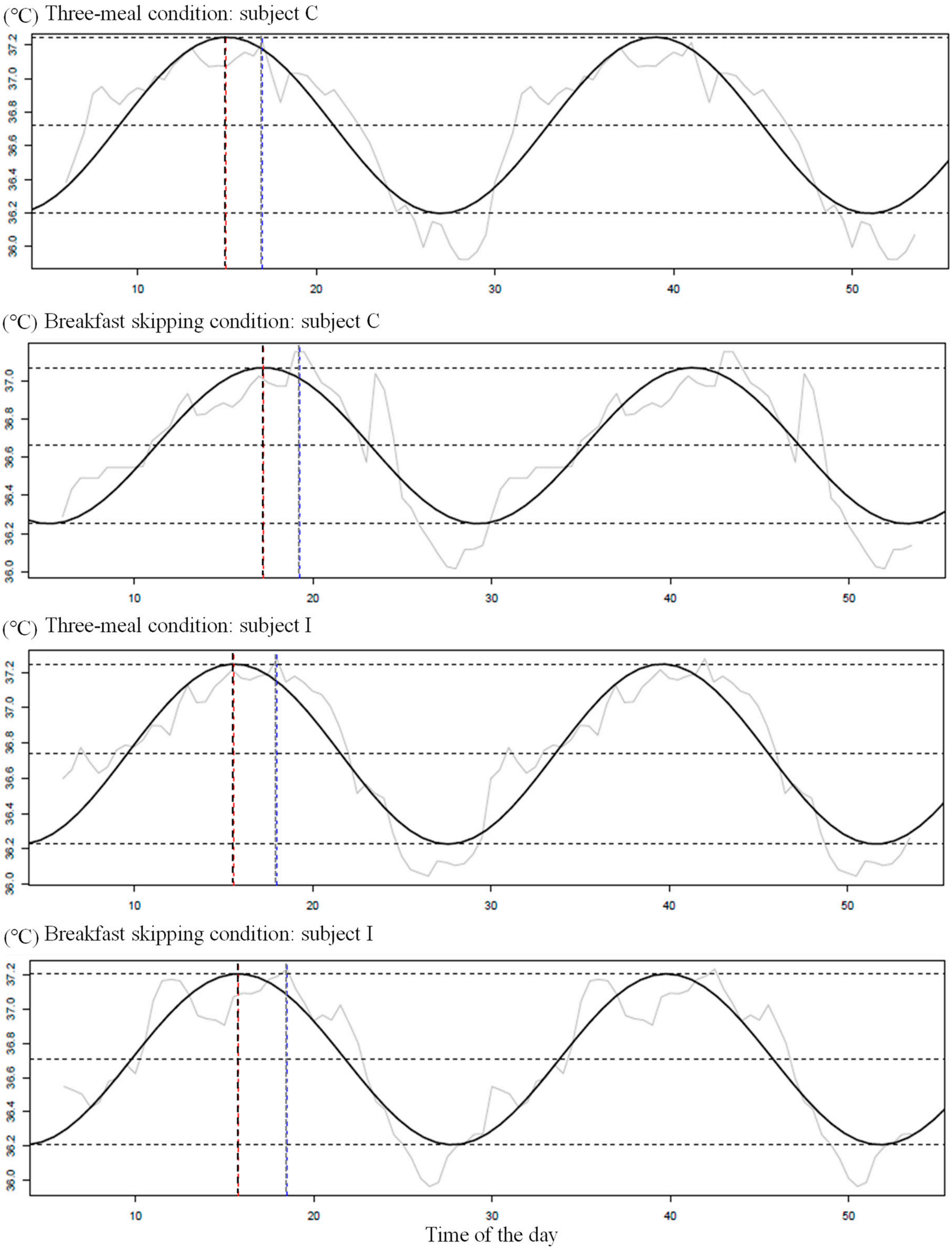

Supplemental Figure 1. Typical example of core body temperature and the results of the cosinor analysis. Double plots of the value for core body temperature (thin line) and the results of cosinor fitting (solid line) in the 2 dietary conditions. Peak time is plotted by the broken line.

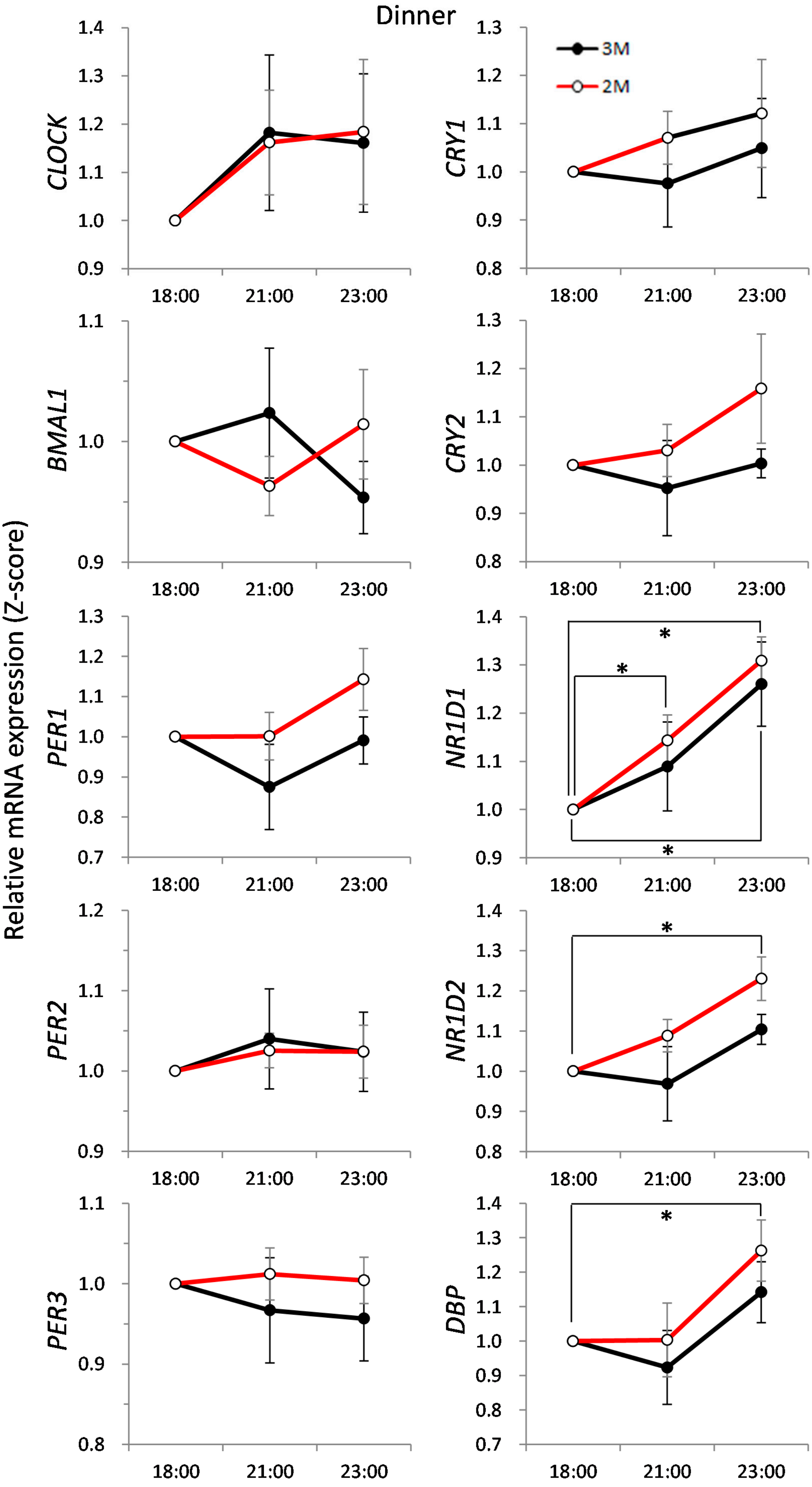
Dinner
3M
2M
Relative mRNA expression (Z-score)
CLOCK
CRY1
BMAL1
CRY2
PER1
NR1D1
PER2
NR1D2
PER3
DBP
18:00
21:00
23:00
*

Supplemental Figure 2. Gene expression after dinner. The gene clock expression profiles are presented as the mean value ± SEM for the 2 dietary conditions. Blood samples were collected 5.5 h after lunch (time point 18:00), 3 h after dinner (21:00), and 5 h after dinner (23:00). The closed circle indicates the 3M condition (●) and the open circle indicates the 2M condition (○). One-way ANOVA was used to evaluate the effect of a meal on expression of the clock gene. As a post-hoc test, multiple comparisons using Dunnett's test were conducted.
